# Supplementary material for: Effects of age and sex on association between toe muscular strength and vertical jump performance in adolescent populations
Source: PLoS One. 2021 Dec 31;16(12):e0262100. doi: 10.1371/journal.pone.0262100 (PMC8719687; doi:10.1371/journal.pone.0262100)
Supplement: Attachment — Submitted filename: Reply PONE-D-21-13635 2nd.docx [file pone.0262100.s002.docx]

Reviewer #1: Thank you for asking me to re-review this paper. I congratulate the authors on reworking the analysis to ensure independence of data, and reworking sections of the manuscript accordingly.

*Thank you very much for your constructive comments We believe that the manuscript has improved significantly through the current revision.*

I have just a few comments

1. Jump height I assume was normalised to body height by the statement on page 10 line 158 that the height recorded was that ‘above the metal pole’. Could you clarify if the metal pole was set at the participants stretched arm height, or something else?

*We are very sorry that we misunderstood your previous comments. According to your comments, we added the sentence as follows,*

*“Before the jump test, the participants were required to stand near the tester and raise their dominant hand to displace the plastic vanes for adjusting the effect of the participant’s height.”*

2. Please include the interpretation you used for the correlation, and reference it. The criteria used should be reported in the methods statistical section, the corresponding category used in your discussion line 216 (currently ‘relatively weak’), and inserted in your conclusion line 286 to describe the strength of the association. E.g TGS is relatively weakly associated with….

*We added the reference and explanation of the criteria used in the statistical section.*

*We added the term of “relatively weakly” in the conclusion.*

Reviewer #2: Thank you again for your effort on this manuscript! I appreciate the time and consideration taken to make these changes.

*Thank you very much for your constructive comments. We are thankful for the time and energy you expended.*
